# The Influence of Cement Thickness within the Cap on Stress Distribution for Dental Implants

**DOI:** 10.3390/jfb15070199

**Published:** 2024-07-21

**Authors:** Mario Ceddia, Tea Romasco, Luca Comuzzi, Alessandro Cipollina, Adriano Piattelli, Gianna Dipalma, Angelo Michele Inchingolo, Francesco Inchingolo, Natalia Di Pietro, Bartolomeo Trentadue

**Affiliations:** 1 Department of Mechanics, Mathematics and Management, Polytechnic University of Bari, 70125 Bari, Italy; marioceddia1998@gmail.com (M.C.); bartolomeo.trentadue@poliba.it (B.T.); 2 Department of Medical, Oral and Biotechnological Sciences, “G. D’Annunzio” University of Chieti-Pescara, Via dei Vestini 31, 66100 Chieti, Italy; tea.romasco@unich.it; 3 Center for Advanced Studies and Technology (CAST), “G. D’Annunzio” University of Chieti-Pescara, 66100 Chieti, Italy; 4 Independent Researcher, 31020 San Vendemiano, Italy; luca.comuzzi@gmail.com; 5 Independent Researcher, 92019 Sciacca, Italy; alexandros1960@libero.it; 6 School of Dentistry, Saint Camillus International University of Health and Medical Sciences, 00131 Rome, Italy; apiattelli51@gmail.com; 7 Facultad de Medicina, UCAM Universidad Católica San Antonio de Murcia, 30107 Murcia, Spain; 8 Department of Interdisciplinary Medicine, University of Bari “Aldo Moro”, 70124 Bari, Italy; gianna.dipalma@uniba.it (G.D.); angeloinchingolo@gmail.com (A.M.I.); francesco.inchingolo@uniba.it (F.I.)

**Keywords:** prosthetic cement, biomechanics, finite element analysis (FEA), implant stress analysis, dental materials, dental prosthesis, Morse cone, conometric connection

## Abstract

The purpose of this finite element analysis (FEA) was to evaluate the stress distribution within the prosthetic components and bone in relation to varying cement thicknesses (from 20 to 60 μm) utilized to attach a zirconia crown on a conometric cap. The study focused on two types of implants (Cyroth and TAC, AoN Implants, Grisignano di Zocco, Italy) featuring a Morse cone connection. Detailed three-dimensional (3D) models were developed to represent the bone structure (cortical and trabecular) and the prosthetic components, including the crown, cement, cap, abutment, and the implant. Both implants were placed 1.5 mm subcrestally and subjected to a 200 N load at a 45° inclination on the crown. The results indicated that an increase in cement thickness led to a reduction in von Mises stress on the cortical bone for both Cyroth and TAC implants, while the decrease in stress on the trabecular bone (apical zone) was relatively less pronounced. However, the TAC implant exhibited a higher stress field in the apical area compared to the Cyroth implant. In summary, this study investigated the influence of cement thickness on stress transmission across prosthetic components and peri-implant tissues through FEA analysis, emphasizing that the 60 μm cement layer demonstrated higher stress values approaching the material strength limit.

## 1. Introduction

Prosthetic restorations currently employ a variety of materials. However, both metal-ceramic and double-layer all-ceramic restorations are prone to technical complications, primarily the cohesive fracture of the veneering ceramic, commonly referred to as chipping [1,2,3,4,5]. The monolithic design of all-ceramic crowns has been suggested as a way to reduce mechanical issues due to their exceptional anti-fracture mechanical properties and biocompatibility [6]. Monolithic zirconia has emerged as an alternative material to decrease the occurrence of mechanical complications associated with the fracture of the veneering ceramic, while also streamlining manufacturing time and enhancing cost-effectiveness [1,6]. Nevertheless, discrepancies in the cementation gap resulting from different zirconia milling procedures can affect the final retention of the crown [7].

When selecting the method for the final restoration of an implant with a prosthetic element, the primary options are typically screwed fixation and cemented fixation. Cemented crowns, while challenging to remove, offer superior aesthetic qualities and higher resistance to fracture compared to screw-fixed crowns. Screw-fixed crowns, on the other hand, are prone to aesthetic issues, premature screw loosening, and crown fractures, despite being easier to remove for maintenance purposes [8,9,10,11,12]. Additionally, the cement layer can act as a shock absorber, transferring occlusal loads to the implant-bone complex [13,14,15,16]. To enhance conventional retention methods, a novel connection involving the use of a coping inserted into the crown and subsequently placed on the abutment has been introduced (biconometric concept) [17]. This innovative connection involves the utilization of a conometric coping, which serves as an intermediary component between the implant abutment and the prosthetic crown. The conometric connection features a male cone on the connecting element (abutment) and a female cone on the prefabricated cap, meticulously designed to interlock securely without the requirement of cement or screws [18]. This approach is based on the friction between the abutment’s outer surface and the coping’s inner surface to establish an effective biological seal and mitigate the risk of bacterial infiltration and peri-implantitis. This approach facilitates the secure attachment of prosthetic components, mitigates the risk of cement residue or screw loosening, and allows for the convenient removal and reinsertion of the prosthesis when necessary. Furthermore, the tapered connection not only ensures stability but also promotes enhanced hygiene by eliminating potential cement-related inflammation and peri-implant complications. The precise fit and the physical phenomenon of friction ensure the prosthesis’ retention [17,19]. It is noteworthy that an increase in the taper angle of the connection reduces system retention, whereas a smaller taper angle increases retention, making disengagement of the connection challenging due to elevated interface forces [20,21]. Furthermore, an in vitro study demonstrated that the retention force remains constant after 5000 cycles of coping insertion and separation, highlighting the overall effectiveness of this taper system [22]. In this type of connection, the choice of cement for the attachment of the prosthetic crown to the coping is pivotal in ensuring the longevity of the prosthetic restoration. The dental industry offers a wide range of cement options, with resin-based and glass ionomer cement being the most commonly used ones [23,24,25,26,27,28,29,30]. Resin cement exhibits superior bonding to enamel surfaces, dentin, and metal alloys compared to glass ionomer cement, along with accelerated setting times, contributing to enhanced crown stability. Resin cement is typically composed of acrylic composites or acrylic resin and adhesive monomers that bond effectively with the substrate [31,32,33,34]. On the other hand, glass ionomer cement, while comparatively less robust and adhesive, offers unique benefits such as the release of fluoride ions, providing effective cavity prevention. These types of cement are often used in scenarios prioritizing cavity protection over mechanical strength, particularly in crown restorations directly onto the tooth [35,36]. 

**Figure 1:**
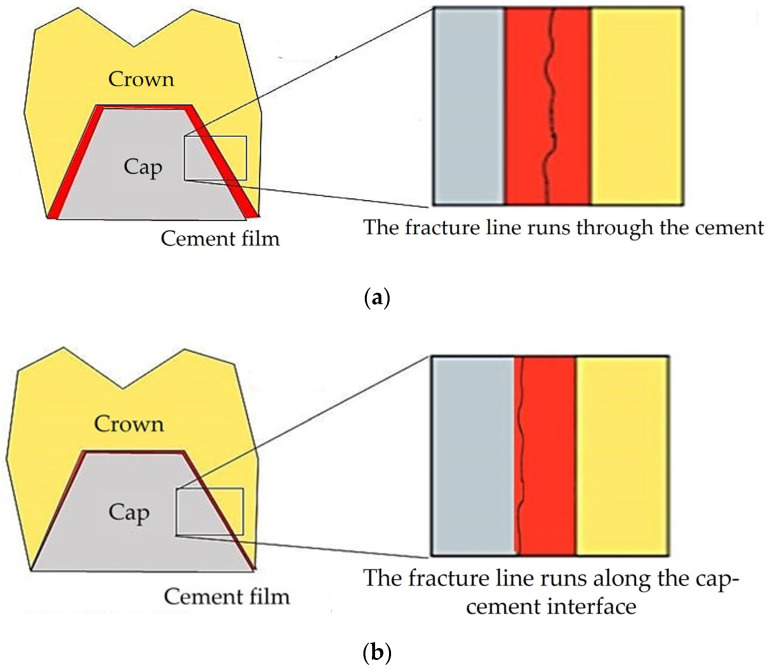
Failure patterns for (**a**) thick and (**b**) thin cement layers between the cap and crown.

The thickness of the adhesive layer between the restoration and the prosthetic element significantly influences the durability of cementation. Thin cement layers promote optimal adhesion and retention of the restoration, while excessively thick cement films can adversely impact retention by propagating cracks and hindering the breaking of robust bonds between the cement and the restoration These cracks advance through the weaker cement layer, surpassing its cohesive strength. Figure 1a shows how an extensive cement thickness results in the fracture line extending into the thick cement layer, which possesses weak cohesive forces and is prone to break first. Conversely, a smaller thickness leads to the extension of the fracture line to the crown–cap interface, where the bond is stronger (Figure 1b). Thus, a thicker layer of cement corresponds to less retention. Moreover, cement film thicknesses exceeding 75 µm can expedite washout and contribute to retention failure [37].

The thickness of cement has a significant impact on stress transmission in both prosthetic components and bone. El-Anwar et al. [37] observed in their study that increasing cement thickness reduced stress on cortical bone. Additionally, other finite element analysis (FEA) studies have examined various cement-related factors influencing stress. These studies revealed that cement with a larger Young’s modulus, indicating stiffer properties, resulted in greater stresses [38,39,40,41]. The numerical methods employed the von Mises criterion to assess stresses in peri-implant tissues and prosthetic components. This criterion is based on the concept that the material begins to fail when the combination of principal stresses exceeds a specific critical value, known as the tensile strength of the material [41].

This study aimed to evaluate stresses on prosthetic components and bone by conducting an FEA on two different implant types and varying the thickness of resin-based cement (20, 40, and 60 µm) on a zirconia crown restoration using a conometric system between the coping and the abutment.

The null hypothesis of this study posited that cement thickness does not significantly influence stress transmission.

## 2. Materials and Methods

### 2.1. Modeling 

**Figure 2:**
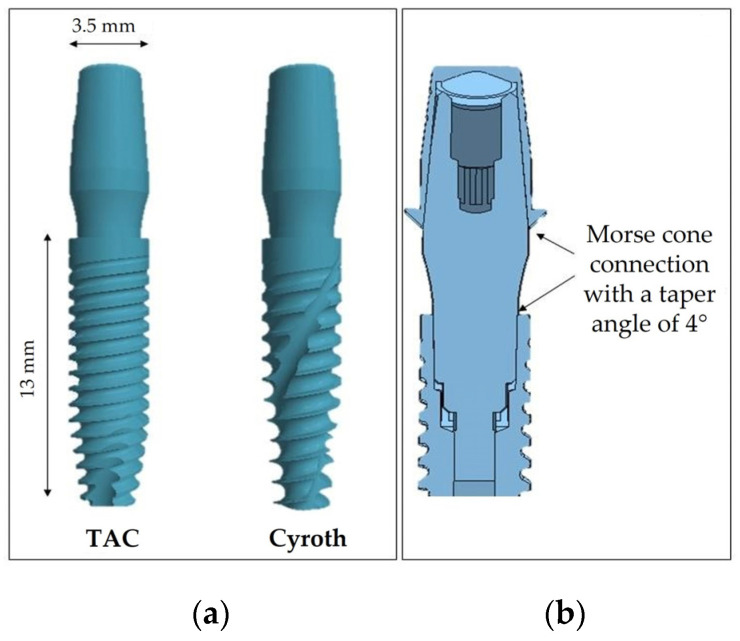
Three-dimensional (3D) models of the implants. (**a**) TAC and Cyroth (AoN Implants, Grisignano di Zocco, Italy) implants; (**b**) cross-sectional view of the implant-abutment and abutment-cap conometric connections.

Two distinct implant macro-morphologies, Cyroth and TAC from AoN Implants, Grisignano di Zocco, Italy, were evaluated (Figure 2a). Both implants are 3.5 mm in diameter and 13 mm in length, presenting a conometric connection between the abutment and coping at a taper angle of 4° and between the fixture and the abutment (Figure 2b). The TAC implant exhibits a tapered and less aggressive collar shape with sharper and more aggressive threads in a single-thread design and a flat implant apex. In contrast, the Cyroth cylindrical implants feature a slightly tapered collar with less aggressive threads designed to compress and deform the bone rather than cut it, as well as a tapered apex.

**Figure 3:**
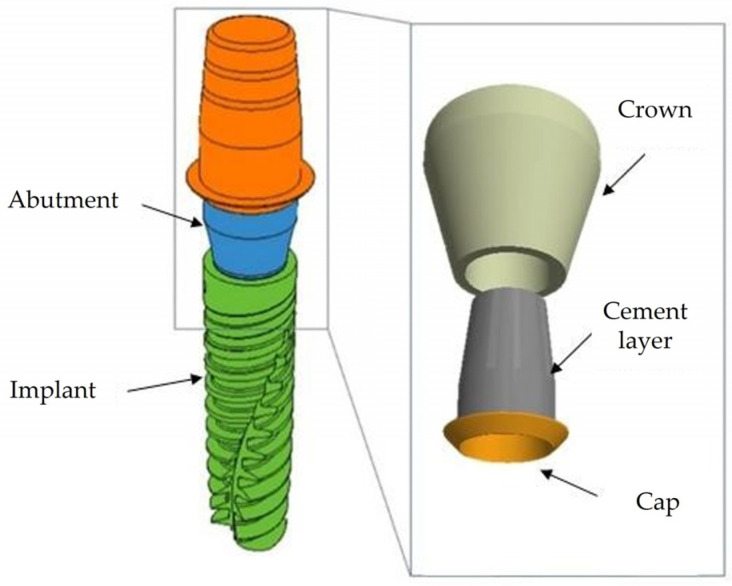
Representative three-dimensional (3D) model of the analyzed components.

Subsequently, the implants were subjected to three-dimensional (3D) modeling using computer-aided design (CAD) software (Autodesk, Inventor 2023.1, San Francisco, CA, USA), as depicted in Figure 3, illustrating the assembly of all components. Following the abutment placement in the implant, the coping was attached with cement, and the crown was inserted with a tapered interface on the abutment. The crown was modeled in a simplified manner, featuring a flat surface in the occlusal contact area. The components provided by AoN Implants (Grisignano di Zocco, Italy) were assembled and bonded using the assemble command in the CAD software.

**Figure 4:**
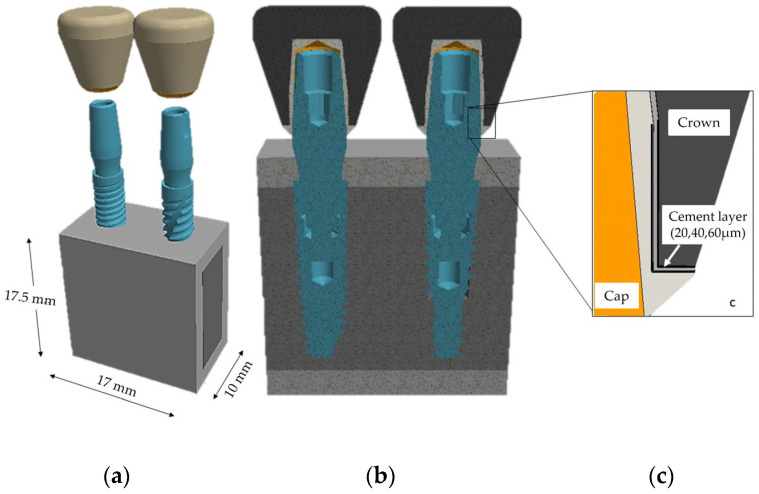
Complete 3D model: (**a**) bone block dimensions; (**b**) cross-sectional representation of the subcrestal implant insertion (1.5 mm); (**c**) cross-sectional detail of the interface between the coping and crown, encompassing varying cement thicknesses (20, 40, 60 μm).

After determining the geometric characteristics of the implants, it was imperative to model the surrounding bone structure. This involved developing a mandibular bone block model based on cross-sectional images of the right first molar from a computed tomography (CT) scan [42]. The thickness of the cortical bone area was measured at 2 mm. To streamline the analysis and reduce computational time, the longitudinal dimension of the bone was extended to 17 mm to accommodate the insertion of two implants (Figure 4a). Subsequently, after assembling the implant components, a hole matching the implant dimensions was created in the bone block to facilitate implant placement. The implants were then inserted subcrestally to a depth of 1.5 mm (Figure 4b). The modeling process also involved examining the impact of three layers of cement (20, 40, 60 µm), as depicted in Figure 4c. 

### 2.2. Material Properties 

This study assumed the isotropic and homogeneous characteristics of the materials, with isotropic behavior indicating consistent mechanical properties in all directions [42,43,44,45,46,47]. Several studies have demonstrated that the mechanical properties of bone are influenced by density [48,49,50]. In this context, bone quality was classified as D1/D2, based on the Misch classification [46]. The coping, abutment, and implant were simulated using a Ti6Al4V titanium alloy, while the crown was modeled using zirconia. The cement employed was a resin-based auto-polymerizing Multilink Hybrid Abutment (MHA) type (Ivoclar Vivadent, Schaan, Liechtenstein), as detailed in Table 1 [51].

Conversely, Table 2 provides an overview of the fundamental mechanical properties designed for utilization in the FEA simulation. Specifically, Young’s modulus (E) defines the material’s stiffness, while Poisson’s ratio (*ν*) characterizes the elasticity of an elastic solid under various loading conditions [42,51,52,53].

### 2.3. Constraints and Loading Conditions

**Figure 5:**
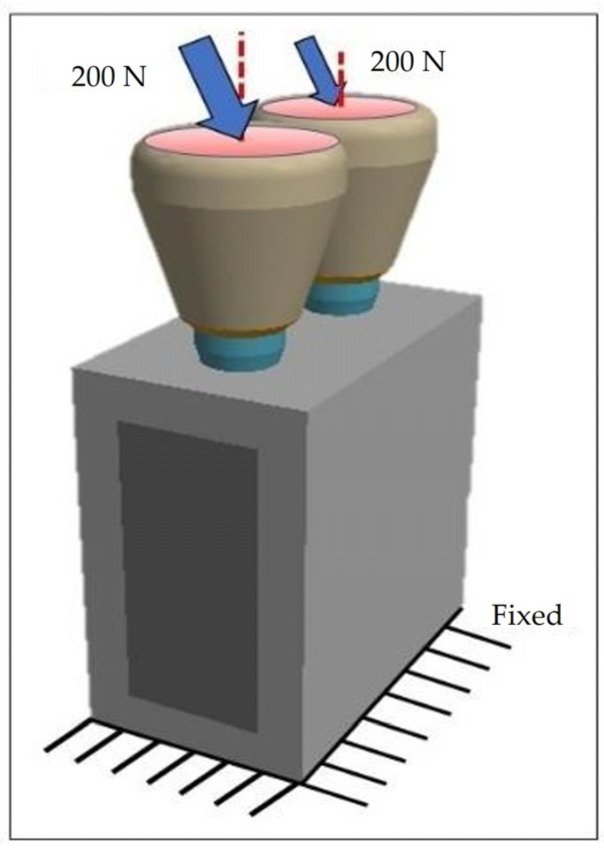
Loading and constraint conditions.

The lower and lateral surfaces of the cortical bone block were constrained from movement in all directions. The loading conditions involved the application of a 45° inclined load relative to the implant’s apical direction, with a force of 200 N on the upper surface of the zirconia crown (highlighted in red), as depicted in Figure 5 [37]. Moreover, the simulation encompassed full contact between cancellous and cortical bone and the implant, aiming to replicate complete osseointegration and simulate realistic conditions at the bone-implant interface. The interaction between the implant, abutment, and coping was designated as a rigid contact to simulate a stable connection without any movement between the components.

### 2.4. Finite Element Analysis (FEA)

**Figure 6:**
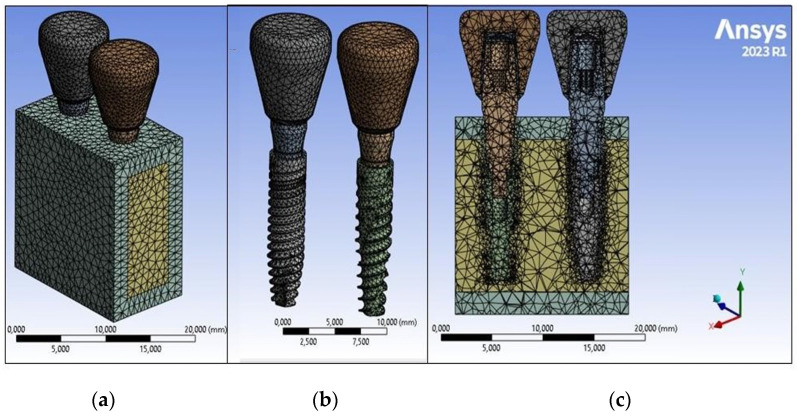
Model meshes: (**a**) mesh of the entire model; (**b**) mesh of the two implants; (**c**) mesh of the sectional view of the model.

Finite element modeling was conducted using FEA software (ANSYS 2023 R1, Workbench, Canonsburg, PA, USA). The implant was removed from the bone model using volume subtraction to create the implant cavity. Subsequently, the implant was carefully fitted into the bone block to replicate complete osseointegration. All models were discretized into solid elements (Solid 45) with three degrees of freedom in all axes [37]. A sensitivity analysis was performed to determine the optimal mesh size for minimizing stress result errors. A 0.5 mm mesh size was chosen based on a 2% minimum error, consistent with previous findings in the literature [19,43,46] (Figure 6). Subsequently, a total of 87,033 elements and 152,096 nodes were generated for all 3D models as a result of this analysis.

In the course of static analysis, a computer with an Intel Core i7 processor operating at 2.90 GHz and 16 GB of RAM was utilized. Following the implementation of the 3D model in FEA software (ANSYS 2023 R1, Workbench, Canonsburg, PA, USA), the von Mises stress values and distributions were examined. Stress distribution was visually represented using color maps, where red zones indicated the highest values and blue zones denoted the least critical areas. Subsequently, stress and strain values were evaluated at different points within the models and then compared.

## 3. Results

The outcomes resulting from computational processing using FEA software (ANSYS 2023 R1, Workbench, Canonsburg, PA, USA) were utilized to analyze von Mises stresses within cortical and trabecular bone tissues as well as implants. 

### 3.1. Stress Analysis on Bone

**Figure 7:**
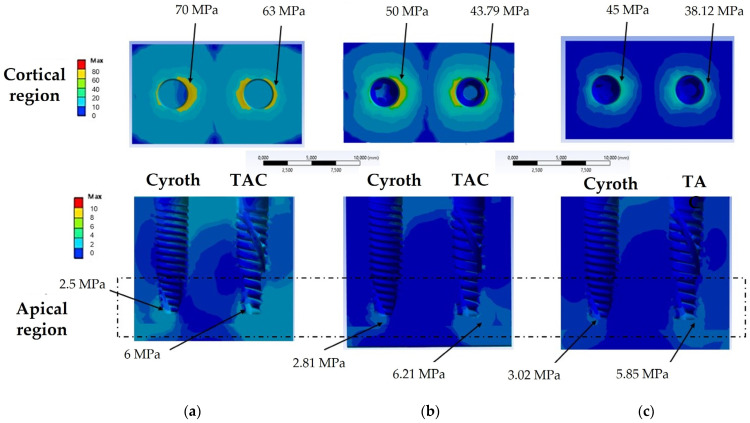
The von Mises stress analysis on bone testing a cement layer of (**a**) 20 µm, (**b**) 40 µm, and (**c**) 60 µm.

Figure 7 presents the von Mises stress findings in the cortical and apical regions of bone, using 20, 40, and 60 µm cement layer thicknesses, respectively.

Figure 7a shows that the maximum von Mises stress value in the cortical bone at the implant contact zone was 70 MPa for the Cyroth implant and 63 MPa for the TAC implant, with both implants having a 20 µm thick cement layer. Moreover, the TAC implant demonstrated an elevated stress of 6 MPa at the apical zone, in contrast to 2.5 MPa for the Cyroth at the same zone.

In the presence of a 40 µm thick cement layer, a reduction in stress within the cortical zone was observed. Specifically, the reported stress values were 50 MPa for the Cyroth implant and 43.79 MPa for the TAC implant. In the apical zone, the values were 2.81 MPa for the Cyroth implant and 6.21 MPa for the TAC implant (Figure 7b).

In the case of the Cyroth implant, a cement thickness of 60 µm resulted in a cortical bone stress of 45 MPa, whereas the TAC implant recorded 30.12 MPa. In the apical zone, the Cyroth implant experienced a stress of 3.02 MPa, while the TAC implant was 5.85 MPa (Figure 7c).

**Figure 8:**
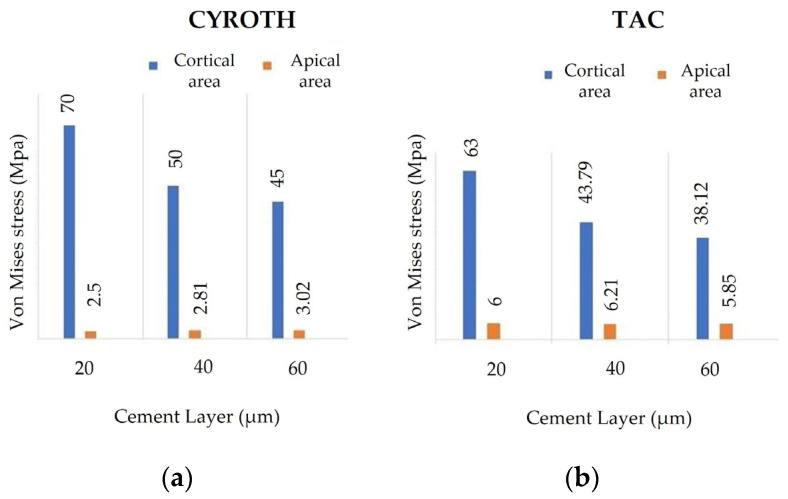
The von Mises stress results for (**a**) Cyroth and (**b**) TAC implants, considering various cement layers (20, 40, and 60 µm).

The data presented in Figure 8 summarize the von Mises stress outcomes within the bone when utilizing the two implants while considering various cement thicknesses (20, 40, and 60 µm). In the cortical zone, it was observed that stress levels were reduced for both implants as the cement thickness increased. The stress distribution within the apical zone exhibited a consistent trend. Furthermore, it was noted that the TAC implant induced higher stress in the apical region compared to the Cyroth implant for all considered cement thicknesses. This behavior can be explained by the design of the TAC implant, characterized by a more tapered thread shape in the apical region, resulting in a different force concentration compared to implants with wider threads. This variance directly impacts the stress distribution within the bone and its capacity to endure such loads.

### 3.2. Stress Analysis on the Implants

**Figure 9:**
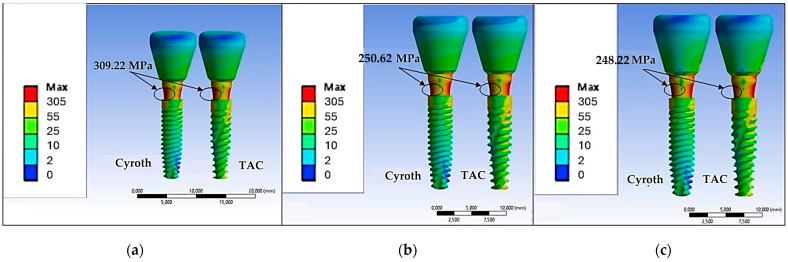
The von Mises stress values on prosthetic components with various cement thicknesses: (**a**) 20 µm, (**b**) 40 µm, and **(c**) 60 µm.

Similarly, an increase in cement thickness resulted in a corresponding reduction in the maximum stress on the abutment neck at the point of contact with the implant (Figure 9).

The stress zones identified in this scenario are deemed reasonable as the application of a 200 N load at a 45° angle to the prosthetic crown resulted in bending on the abutment, leading to the localization of stresses in that specific area. Following the examination of Figure 9 b,c, it is evident that the stress reduction became less sensitive for cement thicknesses between 40 and 60 µm. Specifically, with a cement thickness of 20 µm, the maximum stresses at the abutment for both implants were approximately 309.22 MPa. Increasing the thickness to 40 µm reduced the maximum stress to about 250.62 MPa, which stabilized at around 248.22 MPa with a 60 µm cement thickness. While the stress on the abutments was found to be comparable for both the Cyroth and TAC implants, there was a notable difference in the stress distribution on the implant bodies. Figure 9 illustrates that the TAC implant experienced higher stress levels due to its more tapered morphology. This is evident in the increased yellow areas observed on the TAC implant.

### 3.3. Stress Analysis on the Cement

**Figure 10:**
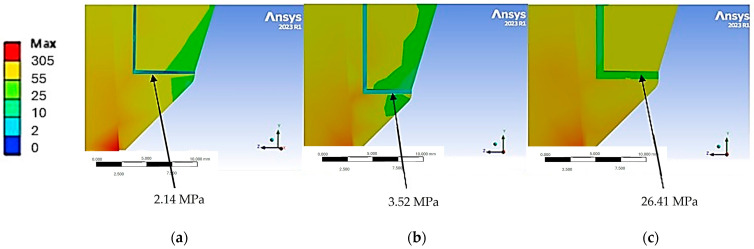
Maximum von Mises stress values on cement based on its thickness: (**a**) 20 µm, (**b**) 40 µm, and (**c**) 60 µm.

Figure 10 shows the von Mises stress distribution between the cement and the crown and coping prosthetic structures. 

Upon increasing the thickness of the cement, a corresponding increase in internal stress was observed. This phenomenon explains the rationale for the stiffer system behavior observed when employing a thin layer of cement, with the majority of stress being distributed to the implant and bone. Conversely, an increase in thickness resulted in the absorption of stress distribution. Notably, a 60 µm layer exhibited a higher load absorption by the cement at 26.41 MPa, in contrast to the 2.41 MPa load absorption with a reduced thickness of 20 µm. It is important to note that the optimal strength for a resin-based cement is approximately 29.7 MPa and exceeding the 60 µm thickness threshold may lead to potential strength issues [37].

## 4. Discussion

The main objective of this study was to assess the stress applied to prosthetic components and bone using the FEA of two distinct types of implants. The investigation involved manipulating the thickness of resin-based cement (20, 40, and 60 µm) on a zirconia crown restoration featuring a conometric connection between the coping and the abutment. The findings, in line with the existing literature, indicated that an increase in cement thickness from 20 to 60 µm led to reduced stresses, particularly on cortical bone. Additionally, it was observed that this increase in thickness induced a variation in the internal stress distribution within the cement, resulting in critical mechanical strength phenomena for 60 µm thicknesses. This stress level approached the strength of resin cement. Consequently, the null hypothesis is rejected due to the significant impact on stress transmission within prosthetic structures and peri-implant tissues due to variations in cement thickness.

The choice between cemented and screw-retained prostheses in the field of implant dentistry is a topic of considerable interest within the dental community [13,14,15,54]. Cemented prostheses offer several advantages, including precise fitting, strong biomechanical stability, absence of screw access holes, superior occlusal design, and adaptability to accommodate malpositioned implant prosthetics. Furthermore, the cement layer acts as a shock absorber, compensating for dimensional variations between the restoration and the anchor element. However, a potential issue with cemented prostheses is the difficulty associated with the removal of excess cement, leading to complications such as peri-implantitis, peri-implant mucositis, and marginal bone loss [8,9,10,55]. On the other hand, screw-retained prostheses offer facilitated retrieval but are more susceptible to technical issues, including component fractures and screw loosening [12,56,57]. Moreover, in a cemented restoration, the reduction in the number of screws restricts the micro-movement of the components. While this reduction may be advantageous in addressing issues linked to the mechanical strength of prosthetic components, it also influences the transmission of forces within the implant system. Indeed, the decrease in micro-movements alters stress distribution, particularly on bone structures [58]. A direct transmission of masticatory loads from the occlusal surface to the bone is evident, signifying that forces generated during mastication may not be adequately dissipated by prosthetic components, ultimately leading to heightened stress on bone tissues [58,59].

The challenges associated with existing retention systems in dental prosthetics have led to the introduction of an innovative prosthetic connection called the Morse cone (conometric concept) [60]. This novel connection utilizes a conometric coping to connect the implant abutment to the prosthetic crown [18]. This approach enables the secure attachment of prosthetic components, reducing the risk of cement residue or screw loosening, and allowing for the convenient removal and reinsertion of the prosthesis when needed.

The prosthetic crown, as previously mentioned, is attached to the tapered coping using cement, which plays a pivotal role in the success of dental restorations. In the case of tapered coping connections, the cement’s primary function is to securely bond the prosthetic crown to the coping while maintaining excellent aesthetic properties. The mechanical properties of the cement, including type, thickness, and stiffness, are fundamental not only for ensuring proper retention but also for facilitating load transmission without creating areas of stress concentration that could lead to excessive bone resorption. Resin cement has become increasingly popular in the dental field due to its high compressive and tensile strength, low solubility, and favorable aesthetic properties. These cements are notable for their ability to withstand significant force and stress. Moreover, both in vitro and clinical studies have indicated that resin-composite adhesion can aid in stress distribution and the prevention of crack propagation in ceramic material repairs [37,61,62]. 

In an FEA study conducted by El-Anwar et al. [37], the influence of cement thickness on stress distribution in bone structures was investigated. The findings revealed that increasing the cement layer from 40 to 60 µm resulted in a decrease in the maximum von Mises stress on the cortical bone. This effect was more pronounced for the cortical bone compared to the trabecular bone, where the impact was found to be minimal. Furthermore, it was noted that increasing the cement thickness led to a reduction in the maximum von Mises stress for both glass ionomer and zinc phosphate resin cement, with the specific percentage changes varying depending on the thickness and type of the cement. The general implication of increasing the cement thickness is a more favorable stress distribution, which minimizes the maximum stress on cortical bones with a relatively lesser influence on trabecular bones. However, it is noteworthy that excessively thick layers could potentially lead to “washout”, characterized by cement removal due to external stresses, such as those induced by oral fluids. Notably, the study highlighted those thicknesses exceeding 75 µm accelerated washout and led to retention failure. Consequently, this study utilized the FEA method to examine the mechanical behavior of the resin-based cement used to affix prosthetic crowns on a tapered coping positioned on the abutments of two distinct implants, Cyroth and TAC, each characterized by unique implant shapes.

Some limitations should be highlighted in the current study. The models used in this study may not fully replicate actual human oral conditions. As such, further clinical studies are necessary to validate the findings. This approach also poses challenges related to software familiarity, the influence of configuration parameters on results, and the need for a comprehensive understanding of component behavior. It is essential to recognize that while FEA is a valuable numerical investigation method, it is unable to completely mimic tissue behavior or accurately represent the complexity of the biological field. Additionally, it is prone to potential numerical errors. The study made assumptions about the homogeneity, isotropy, and linear elasticity of all materials, as well as the complete osseointegration between the bone and implants, despite these assumptions being impractical in clinical practice [63]. 

The presented FEA study, notwithstanding its limitations, offers a notable conclusion. It indicates that increasing cement thickness can effectively reduce stress, particularly in cortical bone as opposed to trabecular bone, which exhibits a more uniform stress distribution at the interface with the implant body. This study observed that the Cyroth implant induces a greater stress field on the cortical bone compared to the TAC implant, while the TAC implant generates more stress in the apical zone. Additionally, as the cement thickness increases, the internal stress absorbed by the cement also increases. This study notes that criticalities may arise when the cement thickness reaches approximately 60 µm, as the absorbed stress value approaches the material’s strength value. 

Therefore, based on these findings, it may be suggested that the optimal cement thickness ranges between 40 and 60 µm. To verify the accuracy and clinical relevance of the model, it is essential to conduct in vitro and in vivo experimental studies alongside FEA results. Moreover, future FEA studies should encompass real-life clinical conditions, such as masticatory forces and patient-specific anatomical features, to more accurately replicate the environment in which the dental implant will be subjected to loading.

## Figures and Tables

**Table 1:** Type and chemical composition of cement used in the analysis.

Cement	Type/Curing	Composition
Multilink Hybrid Abutment (MHA)	Resin-based cement/ Auto-polymerization	Dimethacrylate, HEMA ^1^, fillers (barium glass, ytterbium (III) fluoride, spheroid mixed oxides, titanium dioxide), MMA ^2^, PMMA ^3^, dimethacrylates, initiators

^1^ Hydroxyethyl methacrylate, ^2^ methyl methacrylate, ^3^ polymethyl methacrylate.

**Table 2:** List of material properties used in the finite element analysis (FEA).

Model	Material	Young’s Modulus (GPa)	Poisson’s Ratio (*ν*)
Crown	Zirconia	205	0.34
Cement	Resin-based cement (MHA)	6.3	0.25
Cap	Ti6Al4V	110	0.35
Abutment	Ti6Al4V	110	0.35
Implant	Ti6Al4V	110	0.35
Jawbone 1	Spongy	1.37	0.30
Jawbone 2	Cortical	13.7	0.30

## Data Availability

All experimental data to support the findings of this study are available from the corresponding author upon request.
